# Detecting progressing events in multiple sclerosis clinics in real-time is possible: Commentary

**DOI:** 10.1177/13524585251409579

**Published:** 2026-01-14

**Authors:** Anneke van der Walt

**Affiliations:** Department of Neuroscience, School for Translational Medicine, Monash University, Melbourne, VIC, Australia; MS and Neuroimmunology Service, Alfred Health, Melbourne, VIC, Australia

Early detection of disease progression in multiple sclerosis (MS) is critical for optimising treatment and improving long-term outcomes. But can this progression be detected in *real time* – during a routine clinical visit, rather than retrospectively? In this edition of *MSJ Controversies*, Zhu and Butzkueven argue that in many clinics, it already is. Drawing on data from MS registries such as MSBase,^1^ which now house over a million Expanded Disability Status Scale (EDSS) scores, they show that real-time detection of progression is not a theoretical ideal but a reality – particularly when serial scores are collected consistently and are accessible at the point of care. In these settings, clinicians can confidently identify confirmed disability worsening (CDW), including both relapse-associated worsening (RAW) and progression independent of relapse activity (PIRA).

However, Voigt and Ziemssen challenge this notion on two critical fronts. First, they note that the biological underpinnings of MS progression – compartmentalised inflammation, axonal degeneration, synaptic loss – often unfold insidiously. Neuroplasticity and fluctuations in functional reserve can mask decline, making progression difficult to detect in a single snapshot. Second, they highlight the system-level gaps: not all clinics have the infrastructure, staff, or digital tools to support structured longitudinal monitoring.^2^

This debate reveals a deeper tension. Is ‘real-time’ detection defined as *instant certainty* during a single visit, or as the *timely recognition* of change using structured data at the point of care? If the latter, then real-time detection is already feasible – and should be the standard. But for many clinicians, especially those in underresourced or high-throughput settings, even basic tools such as EDSS or the Timed 25-Foot Walk are applied inconsistently. Digital tools are emerging – smartphone-based cognitive tools, remote gait analysis, patient-reported outcomes – but remain poorly integrated into routine practice.^3,4^

What’s clear is that progression detection must move beyond retrospective realisation. Clinicians need tools that are not just accurate, but usable – validated, streamlined, and embedded in clinical workflows. This means investing in longitudinal data capture, encouraging adoption of simple functional tests, and developing patient-friendly digital apps that can bridge clinic and home.

Importantly, patient participation must be central. Individuals living with MS often detect subtle changes before clinicians do. Empowering them with tools to track symptoms, mobility, cognition, and fatigue in their own environments will enrich clinical interpretation and promote earlier intervention.^5^

Both sides of the debate agree that early identification of progression is critical. What differs is how we define ‘real time’ and whether we are willing to invest in systems that support it. In this sense, real-time detection is less about momentary certainty and more about *clinical readiness* – the ability to act on meaningful change when it happens. Progression detection in MS should not hinge on idealised models but reflect achievable best practice: consistent measurement, accessible data, and timely clinical decision-making.
